# Xenobiotics that affect oxidative phosphorylation alter differentiation of human adipose-derived stem cells at concentrations that are found in human blood

**DOI:** 10.1242/dmm.021774

**Published:** 2015-11-01

**Authors:** Laura Llobet, Janne M. Toivonen, Julio Montoya, Eduardo Ruiz-Pesini, Ester López-Gallardo

**Affiliations:** 1 Departamento de Bioquímica, Biología Molecular y Celular, Universidad de Zaragoza, 50013-Zaragoza, Spain; 2 Instituto de Investigación Sanitaria de Aragón, Universidad de Zaragoza, 50013-Zaragoza, Spain; 3 CIBER de Enfermedades Raras (CIBERER), Universidad de Zaragoza, 50013-Zaragoza, Spain; 4 Fundación ARAID, Universidad de Zaragoza, 50013-Zaragoza, Spain

**Keywords:** OXPHOS, Xenobiotics, Adipocytes

## Abstract

Adipogenesis is accompanied by differentiation of adipose tissue-derived stem cells to adipocytes. As part of this differentiation, biogenesis of the oxidative phosphorylation system occurs. Many chemical compounds used in medicine, agriculture or other human activities affect oxidative phosphorylation function. Therefore, these xenobiotics could alter adipogenesis. We have analyzed the effects on adipocyte differentiation of some xenobiotics that act on the oxidative phosphorylation system. The tested concentrations have been previously reported in human blood. Our results show that pharmaceutical drugs that decrease mitochondrial DNA replication, such as nucleoside reverse transcriptase inhibitors, or inhibitors of mitochondrial protein synthesis, such as ribosomal antibiotics, diminish adipocyte differentiation and leptin secretion. By contrast, the environmental chemical pollutant tributyltin chloride, which inhibits the ATP synthase of the oxidative phosphorylation system, can promote adipocyte differentiation and leptin secretion, leading to obesity and metabolic syndrome as postulated by the obesogen hypothesis.

## Abstract (precis)

**Summary:** Some medical drugs and environmental chemical pollutants acting on the oxidative phosphorylation system can alter adipocyte differentiation and adipogenesis and, thus, have important consequences for human health.

## INTRODUCTION

The white adipose tissue (WAT) stores energy as triglycerides and secretes endocrine factors such as adipokines. Most WAT mass or volume consists of mature adipocytes. In adult humans, the number of these cells is remarkably stable because new adipocytes differentiate constantly to replace lost adipocytes (Spalding et al., 2008).

*In vitro* differentiation of 3T3-L1 mouse preadipocytes, one of the best characterized models for studying adipogenesis (Green and Kehinde, 1975; Kita et al., 2009), is accompanied by a remarkable increase in the expression of numerous mitochondrial proteins (Newton et al., 2011). The increased basal rate of oxygen consumption in adipocytes compared with preadipocytes could be a clear manifestation of augmented biogenesis of the oxidative phosphorylation (OXPHOS) system during the adipogenic process (Wilson-Fritch et al., 2003). The levels of assembled OXPHOS complexes and mitochondrial DNA (mtDNA) copy number also increase with the adipocyte differentiation of 3T3-L1 cells (Ryu et al., 2013; Shi et al., 2008).

Genetic manipulation of the OXPHOS system can affect adipocyte differentiation. Mouse preadipocytes that overexpress mesodermal developmental transcription factor Tbx15 have reduced mitochondrial mass, basal respiration and mtDNA gene expression. These cells show an impaired differentiation to adipocytes and reduced triglyceride accumulation (Gesta et al., 2011). Mouse adipose tissue-derived stem cells (mASCs) that lacked the CR6/gadd45-interacting protein Gadd45gip1/Crif1, a translation/assembly factor for mtDNA-derived polypeptides, express lower levels of mtDNA-encoded OXPHOS subunits and display disrupted adipocyte differentiation (Ryu et al., 2013). The expression of mitochondrial transcription factor A (TFAM), a key factor for mtDNA transcription and replication, is increased with adipogenic differentiation of human mesenchymal stem cells (hMSCs). When TFAM expression is suppressed in hMSCs with small interfering RNA, expression of the gene encoding NADH-ubiquinone oxidoreductase chain 2 (*MT-ND2*) is lowered and adipogenic differentiation, as determined by adiponectin mRNA levels, is inhibited (Zhang et al., 2013).

In addition to genetic intervention, physiologic or pharmacologic manipulation of OXPHOS can also affect adipogenic differentiation. Oxygen is the ultimate electron acceptor in the OXPHOS electron transport chain (ETC) and hypoxia (1% O_2_) restrains adipogenic differentiation in both mouse preadipocytes and hMSCs (Carriere et al., 2004; Zhang et al., 2013). Nucleoside reverse transcriptase inhibitors (NRTIs) used in human immunodeficiency virus (HIV) treatment inhibit mtDNA polymerase-γ, the enzyme required for mtDNA replication. This provokes mtDNA depletion and decreases adipogenesis in mouse and human preadipocytes (Caron et al., 2004; Stankov et al., 2010, 2013, 2008; Walker et al., 2006).

Numerous chemical compounds used in medicine, agriculture or other human activities can affect OXPHOS function (Degli Esposti, 1998; Nadanaciva and Will, 2011; Wallace, 2008). Because OXPHOS appears to be important for adipocyte differentiation, xenobiotics could also alter adipogenesis. However, xenobiotics are very frequently tested at high concentrations that would never be found in humans. Therefore, we have investigated the effects on adipogenic differentiation of some OXPHOS xenobiotics at low concentrations. Our results support the view that OXPHOS xenobiotics might interfere with mitochondrial ETC and alter adipogenic differentiation in concentrations already reported present in human circulation.

**TRANSLATIONAL IMPACT**

**Clinical issue**

Adipogenesis, the process by which adipocytes are generated from precursor cells, requires high levels of cellular energy. In line with this, an increase in biogenesis of the oxidative phosphorylation system (OXPHOS) is observed in differentiating preadipocytes. Genetic manipulation of OXPHOS is known to negatively affect adipocyte differentiation in model systems, and defects in OXPHOS are associated with a wide range of human multisystem disorders, reflecting its importance in cellular metabolism. Unfortunately, this system is often an unintended target of drug therapies and is responsible, at least in part, for the dose-limiting side effects associated with a large array of pharmaceuticals. Moreover, OXPHOS is frequently the target of many environmental toxins (xenobiotics). In general, the effects of xenobiotics on OXPHOS have been assessed by applying high concentrations that would not be found in humans. Here, the authors evaluate the effects on cell differentiation and function of OXPHOS-targeting xenobiotics at concentrations that have been reported in human circulation.

**Results**

Isolated human adipose tissue-derived stem cells (hASCs) were used to model adipocyte differentiation. As proof-of-principle, the authors first showed that OXPHOS biogenesis is enhanced during the differentiation of these cells. They then evaluated the effect of different xenobiotics, using concentrations that are usually found in human blood. Because some essential polypeptides for OXPHOS biogenesis are mitochondrial DNA (mtDNA)-encoded, the team tested compounds that could interfere with mtDNA replication or translation. Inhibitors of mtDNA replication, including nucleoside reverse transcriptase inhibitors used in the antiretroviral therapy of individuals with AIDS, and inhibitors of mtDNA translation, including ribosomal antibiotics used to fight bacterial infections, were found to decrease cell triglycerides and leptin secretion. On the other hand, an inhibitor of the OXPHOS ATP synthase, the environmental chemical pollutant tributyltin chloride, increased triglyceride levels and leptin discharge.

**Implications and future directions**

This work supports previous studies that indicate that OXPHOS biogenesis is important for adipocyte differentiation. Clinical drugs that reduce mtDNA gene expression, such as inhibitors of mtDNA replication or translation, decrease OXPHOS biogenesis and alter adipogenesis, even at low concentrations. Exposure to environmental toxins that modify OXPHOS complex activities can also interfere with adipocyte differentiation. This finding lends weight to the obesogen hypothesis, in which it is proposed that environmental contaminants have contributed to the increase in obesity and related metabolic disorders in recent years. Important OXPHOS components are mtDNA-encoded. These polypeptides, by themselves or after their interaction with different xenobiotics, modify OXPHOS capacity. Thus, mtDNA genetic variability might also affect adipocyte differentiation and related phenotypes, with potentially pathological consequences.

## RESULTS

### Characterization of human adipose tissue-derived stem cells

**Fig. 1.**
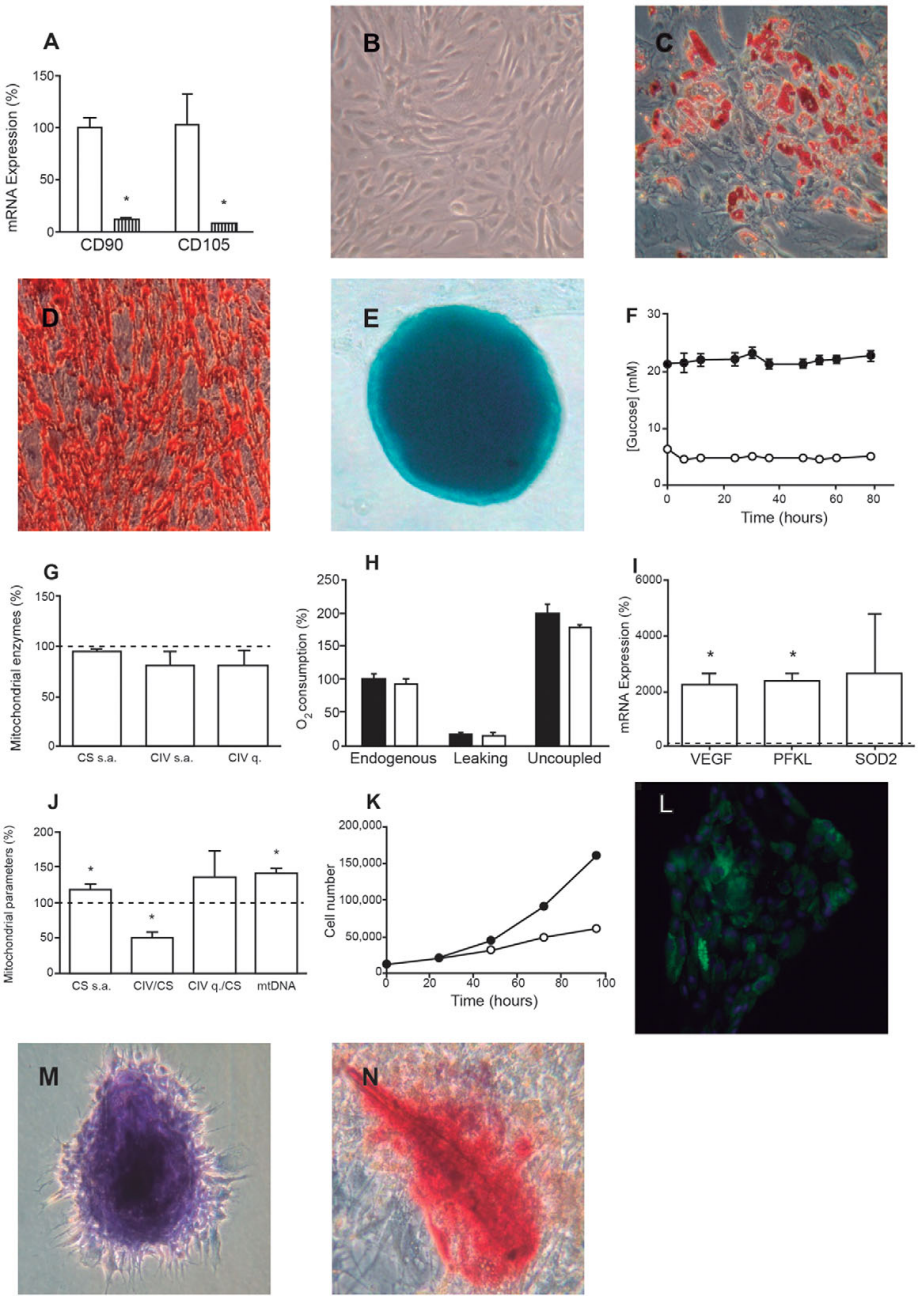
**hASC characterization.** (A) Expression of hASC marker mRNAs. White bars represent hASCs. Striped bars represent neuroblastoma SK-N-BE(2)-C cells, as a negative control. *P≤0.0044. (B-E) Representative microscopy images of hASCs (B), hASCs differentiated to adipocytes (Oil Red) (C), hASCs differentiated to osteocytes (D) and hASCs differentiated to chondrocytes (micromass culture) (E). (F) Glucose concentration of hASC culture medium. Black and white circles indicate 25 and 5 mM glucose, respectively. (G) Mitochondrial enzymes specific activities (s.a.) and levels (q). White bars indicate the value of enzymatic parameters of cells grown at 5 mM glucose. Dotted line represents the value of these variables for cells grown at 25 mM glucose. (H) Oxygen consumption of cells grown at 25 mM (black bars) and 5 mM (white bars) glucose. (I) Expression of hypoxia (3% O_2_) mRNA markers. Dotted line at 100% represents the value of these variables at 20% oxygen. *P≤0.0162. (J) Effect of hypoxia on mitochondrial variables. Dotted line represents the value of these variables at 20% oxygen. *P≤0.0178. (K) hASC doubling time. Black or white circles indicate normoxia or hypoxia, respectively. (L-N) Representative optical microscopy images of hASCs differentiated to adipocytes (Nile Red) (L), chondrocytes (normal culture) (M) and osteocytes (N) in hypoxia.

**Fig. 2.**
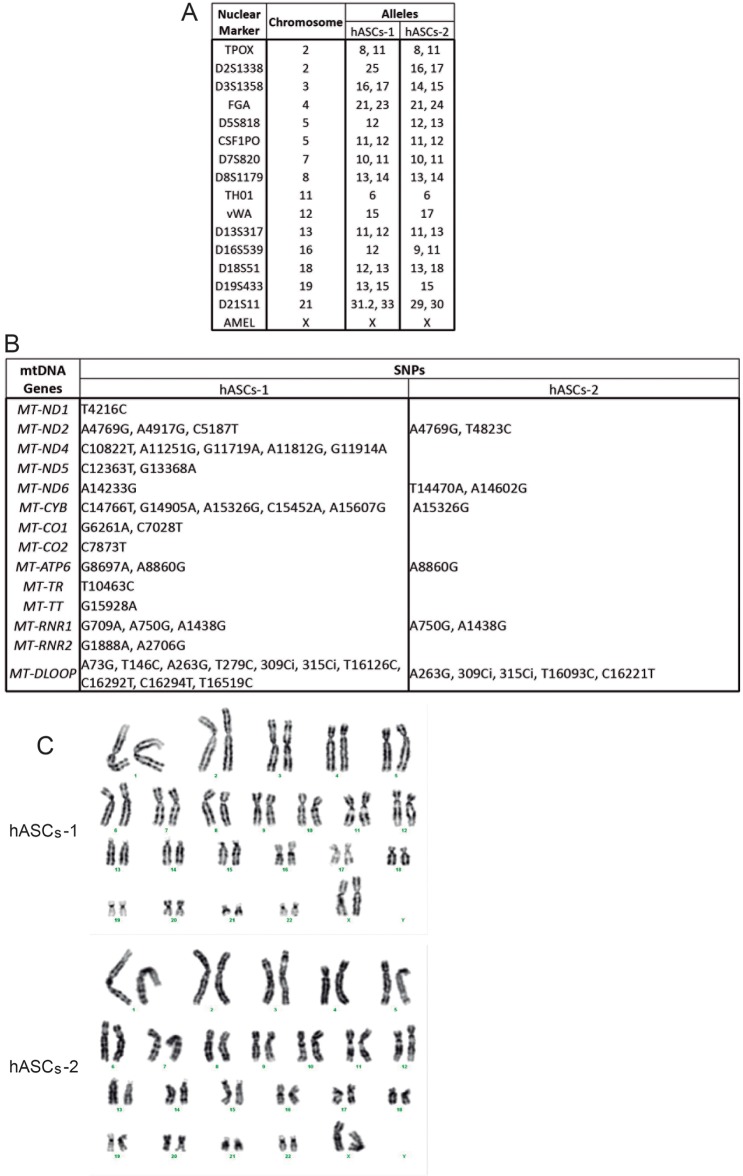
**hASC genetic characterization.** (A) Nuclear genetic fingerprints. (B) mtDNA genetic variants. Sequences are compared with the revised Cambridge reference sequence (rCRS). (C) Karyotypes.

Prior to exposing the cells under study to xenobiotics, they were confirmed to display characteristics typical of human adipose tissue-derived stem cells (hASCs). As previously described (Gir et al., 2012), hASCs were found to adhere to plastic, express mRNAs for the hASC marker proteins cluster of differentiation 90 (CD90/THY1) and 105 (CD105/ENG) (Fig. 1A) and possess the capacity to undergo adipogenic, osteogenic and chondrogenic differentiation (Fig. 1B-E). To genetically characterize these cells, the nuclear genetic fingerprint of 16 short tandem repeats and the whole mtDNA sequence were obtained (Fig. 2A,B). Two different lots of hASCs were used (hASCs-1 and hAScs-2), originating from different donors. The mtDNA haplogroup of hASCs-1 was T2c1d1a and that of hASCs-2 was H10e2. In the organelle genomes, there were no private mutations and none of the genetic variants was apparently pathologic (GenBank KR816716, KR816717) (van Oven and Kayser, 2009). Because all the experiments described here were completed before passage 10, karyotype analysis of the hASCs was performed in this passage. All 20 metaphase preparations were found to be normal 46,XX (Fig. 2C).

It has been previously shown that the glucose concentration in the culture medium can affect different mitochondrial parameters (Weber et al., 2002). We observed that very glycolytic tumor cell lines, such as osteosarcoma 143B cybrids, consumed all medium glucose when grown at low (5 mM) glucose concentration for 48 h (Fig. S1A). Compared with cells grown without glucose, they showed significantly lower quantities of respiratory complex IV (CIV) (Fig. S1B). At high (25 mM) glucose concentration, the sugar concentration in the medium was greatly decreased after 48 h. CIV specific activity and quantity were significantly lower at high glucose than those from cells growing without glucose. The CIV quantity and specific activity of citrate synthase (CS), a volumetric marker of cell fraction occupied by mitochondria, were also significantly lower at high glucose than in cells growing at low glucose. In contrast, glucose consumption by hASCs was almost negligible over 72 h (Fig. 1F). It is possible that hASCs preferably use other substrates, such as pyruvate or glutamine, as energy source (Reitzer et al., 1979). As found in other studies (Keuper et al., 2014), mitochondrial parameters such as CS specific activity, CIV specific activity or quantity (Fig. 1G) and oxygen consumption (Fig. 1H) were not affected by glucose in hASCs.

Several reports have proposed the use of hypoxia for maintaining undifferentiated stem cell cultures, but it is known that hypoxia can also stimulate differentiation (Abdollahi et al., 2011). At 3% oxygen, the mRNA levels of hypoxia markers phosphofructokinase L (PFKL) and vascular endothelium-derived growth factor (VEGF) significantly increased (Fig. 1I) (Lee et al., 2009), as did the number of mtDNA copies and CS specific activity (Fig. 1J), suggesting mitochondrial biogenesis. However, even though CIV quantity tended to increase, CIV specific activity significantly decreased in hypoxia. The hASC doubling time in hypoxia was 45.8 h as opposed to 26.3 h in normoxia (Fig. 1K). Furthermore, the shift to hypoxic conditions slowed adipogenic differentiation but, surprisingly, chondrogenic and osteogenic differentiation was favored by induction of adipogenic differentiation (Fig. 1L-N) (Choi et al., 2014; Wang et al., 2005).

As a result of the slower hASC proliferation rate, the difficulty to induce adipocyte differentiation and confounding effects of chondro/osteogenesis of hASCs under hypoxia, all differentiation experiments were performed at 20% oxygen. Because of the reported delay in adipogenic lineage commitment of hMSCs after glucose reduction (Lo et al., 2011), we also performed these experiments at 25 mM glucose, in spite of the fact that these conditions do not faithfully replicate the true physiological conditions under which hASCs undergo differentiation *in vivo*.

### Cell morphology and metabolic alterations during adipogenic differentiation

**Fig. 3.**
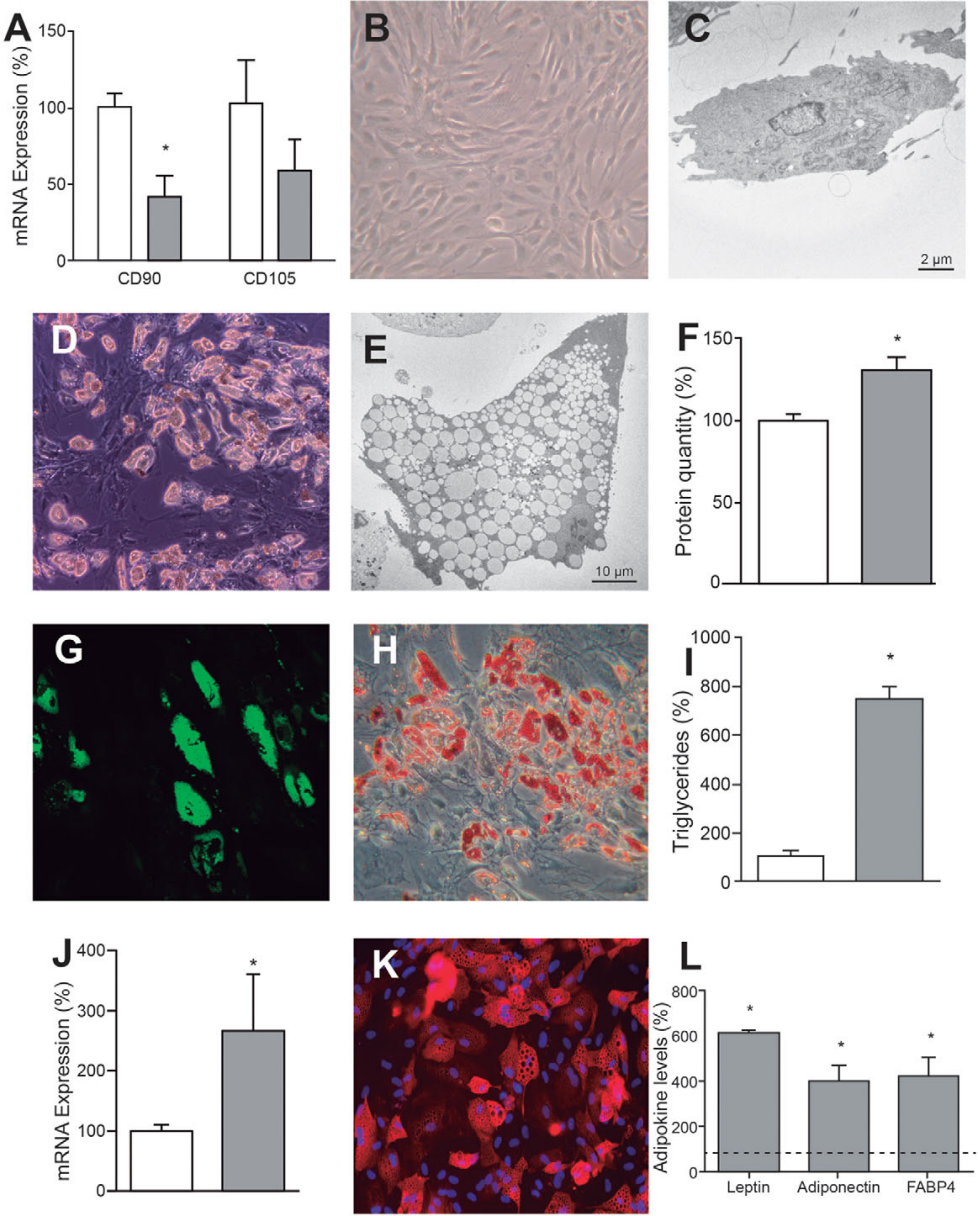
**Adipogenic differentiation.** (A) Expression of hASC marker mRNAs. White and gray bars represent hASCs and adipocytes, respectively. *P=0.0035. (B,C) Representative optical (B) and electron microscopy (C) images of hASCs. (D,E) Representative optical (D) and electron microscopy (D) images of adipocytes. (F) Cell protein amount. White and gray bars represent the protein quantity per million cells of hASCs and adipocytes, respectively. *P=0.0314. (G,H) Representative microscopy images of adipocytes stained with Nile Red (G) or Oil Red O (H). (I) Cell triglyceride levels. White and gray bars represent the quantity of triglycerides in hASCs and adipocytes, respectively. *P<0.0001. (J) Expression of *PPARγ* mRNA. White and gray bars represent hASCs and adipocytes, respectively. *P=0.0442. (K) Representative immunocytochemical image of adipocytes FABP4. (L) Adipokine levels secreted by adipocytes. Dotted line represents the levels found in the hASC culture medium. *P≤0.0117.

As expected, adipogenic differentiation medium slowed hASC proliferation. The mRNA levels of hASC marker proteins CD90 and CD105 decreased by 57.4% and 40.8%, respectively (Fig. 3A). The onset of differentiation was not completely synchronized and, as previously reported (Hofmann et al., 2012; Keuper et al., 2014), a certain percentage of the cells (approximately 50% in our case) did not differentiate at all. Compared with undifferentiated hASCs (Fig. 3B,C), differentiated cells showed a morphological change and increased volume (Fig. 3D,E) which, at times, has been reported to be as high as 20-fold (von Heimburg et al., 2005). The increased size was only partially associated with higher protein content (Fig. 3F) and mainly represented accumulation of lipid droplets, as shown by lipophilic staining (Fig. 3G,H) and triglyceride assay (Fig. 3I). The appearance of lipid droplets is a clear indicator of adipocyte differentiation.

As described in other reports (Gesta et al., 2011; Hofmann et al., 2012; Keuper et al., 2014; Vankoningsloo et al., 2006; Zhang et al., 2013), the induction of adipogenic differentiation led to expression of mRNAs for adipocyte markers adiponectin, leptin, fatty acid binding protein 4 (FABP4), peroxisome proliferator-activated receptor gamma coactivator 1-alpha (PGC1α) and peroxisome proliferator-activated receptor gamma (PPARγ) which, except for PPARγ, were not expressed at quantifiable levels in undifferentiated hASCs (Fig. 3J). Consistently, protein levels for intracellular FABP4 (Fig. 3K) and secreted FABP4, adiponectin and leptin (Fig. 3L) were greatly induced (Dicker et al., 2005).

### Mitochondrial morphology and the OXPHOS system during adipogenic differentiation

**Fig. 4.**
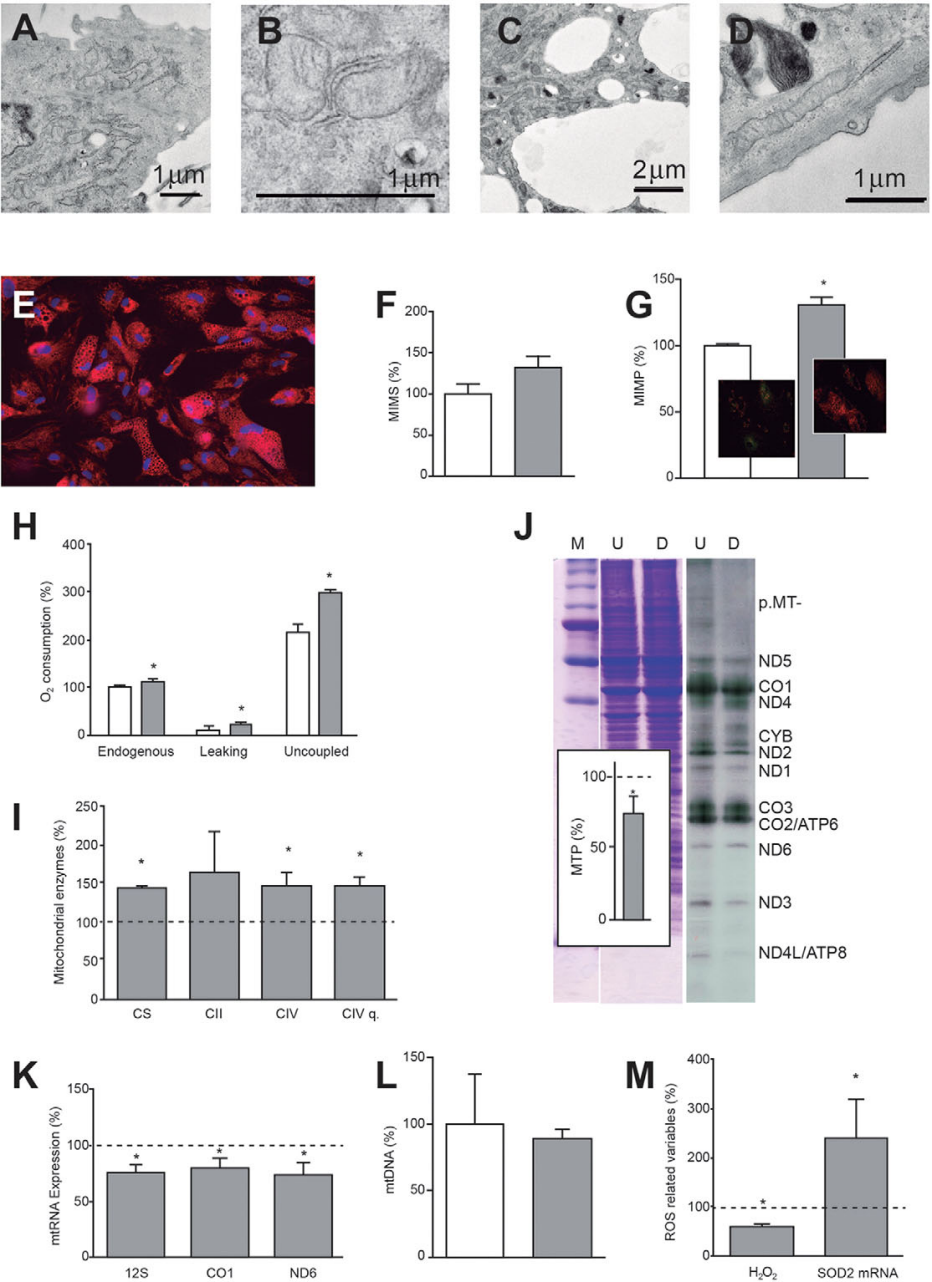
**Mitochondrial changes during adipocyte differentiation.** (A,B) Representative electron microscopy images of part of a hASC (A) and mitochondria (B). (C,D) Representative electron microscopy images of an adipocyte fragment (C) and mitochondrion (D). (E) Representative microscopy image of the mitochondrial disposition in adipocytes. (F) MIMS. White and gray bars represent hASC and adipocyte MIMS values, respectively. (G) MIMP. White and gray bars represent hASC and adipocyte MIMP values, respectively. *P=0.0034. Inserts: representative images of hASCs (left) and adipocytes (right) stained with a dye used to measure MIMP. Green changes to orange when MIMP increases. (H) Oxygen consumption. White and gray bars represent values of consumed oxygen in hASCs and adipocytes, respectively. *P≤0.0361. (I) Mitochondrial enzyme specific activities and quantity (q) in adipocytes. Dotted line represents enzyme activities and amount found in hASCs. *P≤0.0388. (J) Mitochondrial protein synthesis. Representative gel showing the electrophoretic patterns of mitochondrial translation products (ND1-6,CYB, CO1-3 and ATP6,8) (right) and loading controls (left). M, molecular weight marker; U, undifferentiated cells (hASCs); D, differentiated cells (adipocytes). Insert: quantification of mitochondrial translation products (MTPs) in adipocytes. Dotted line represents the level of MTPs in hASCs. *P=0.0210. (K) Mitochondrial transcripts in adipocytes. Mitochondrial RNA levels (12S, CO1 and ND6) represent the three transcription units of the mtDNA. Dotted line represents levels in hASCs. *P≤0.0104. (L) mtDNA levels. White and gray bars represent the mtDNA amount in hASCs and adipocytes, respectively. (M) H_2_O_2_ quantity and *SOD2* mRNA levels in adipocytes. Dotted line represents levels of these parameters in hASCs. *P≤0.0463.

Small, round mitochondria with few cristae are commonly found in small numbers, often in the perinuclear cytoplasm of undifferentiated hASCs (Fig. 4A,B). In adipocytes (Fig. 4C,D), numerous, large and extended mitochondria with multiple cristae are preferentially distributed between the cytoplasmic lipid droplets. These results were confirmed in live cells using a mitochondrial stain (Fig. 4E). Although simple microscopic observation suggested that the mitochondrial inner membrane surface (MIMS) would be larger in differentiated cells, the increase was not found to be significant according to cardiolipin assay (Fig. 4F). This can probably be explained by the fact that only approximately half of the cells used for quantitative assay were differentiated.

The mitochondrial inner membrane potential (MIMP), which drives OXPHOS, was significantly augmented with differentiation (Fig. 4G). The endogenous, leak state and uncoupled oxygen consumption per cell significantly increased with differentiation (Fig. 4H). However, these parameters did not change when normalized by protein amount or CS specific activity. Accordingly, specific activities of CS and CIV, and the quantity of CIV per cell, also increased with differentiation. The increase in respiratory complex II (CII) specific activity was also suggestive (Fig. 4I). Again, these parameters did not change when normalized by CS specific activity. All these biochemical results confirm mitochondrial biogenesis during differentiation. Despite the increase in mitochondrial biogenesis, mitochondrial protein synthesis in differentiated cells was lower than in undifferentiated cells (Fig. 4J). Actively dividing, undifferentiated cells are likely to require continuous mitochondrial protein synthesis to produce mitochondria for the new cells. However, differentiated, non-proliferating cells only need to replace the degraded mitochondrial proteins of existent cells. In agreement with protein levels, the RNA expression for three mtDNA-encoded products (representing three mtDNA transcription units) also decreased with adipocyte differentiation (Fig. 4K), although mtDNA levels remained unaltered (Fig. 4L).

Despite the observed increase in MIMP, the hydrogen peroxide (H_2_O_2_) levels were significantly reduced in differentiated cells (Fig. 4M). This is probably a consequence of increased expression of antioxidant enzymes during adipogenic differentiation (Higuchi et al., 2013). Indeed, it was demonstrated that adipogenesis was accompanied by significantly increased levels of mRNA for manganese superoxide dismutase (SOD2) (Fig. 4M).

### OXPHOS xenobiotics and adipogenic differentiation

#### Nucleoside reverse transcriptase inhibitors: Zidovudine and Zalcitabine

As previously commented, NRTIs, in addition to their antiretroviral effects, inhibit mtDNA polymerase-γ, alter mitochondrial function and can result in disturbed cellular differentiation. Two NRTIs, 3′-azido-3′-deoxythymidine (Zidovudine, AZT) and 2′,3′-dideoxycytidine (Zalcitabine, ddC) were tested here at concentrations close to their maximum reported concentration in human plasma (Gustavson et al., 1990; Laskin et al., 1989).

**Fig. 5.**
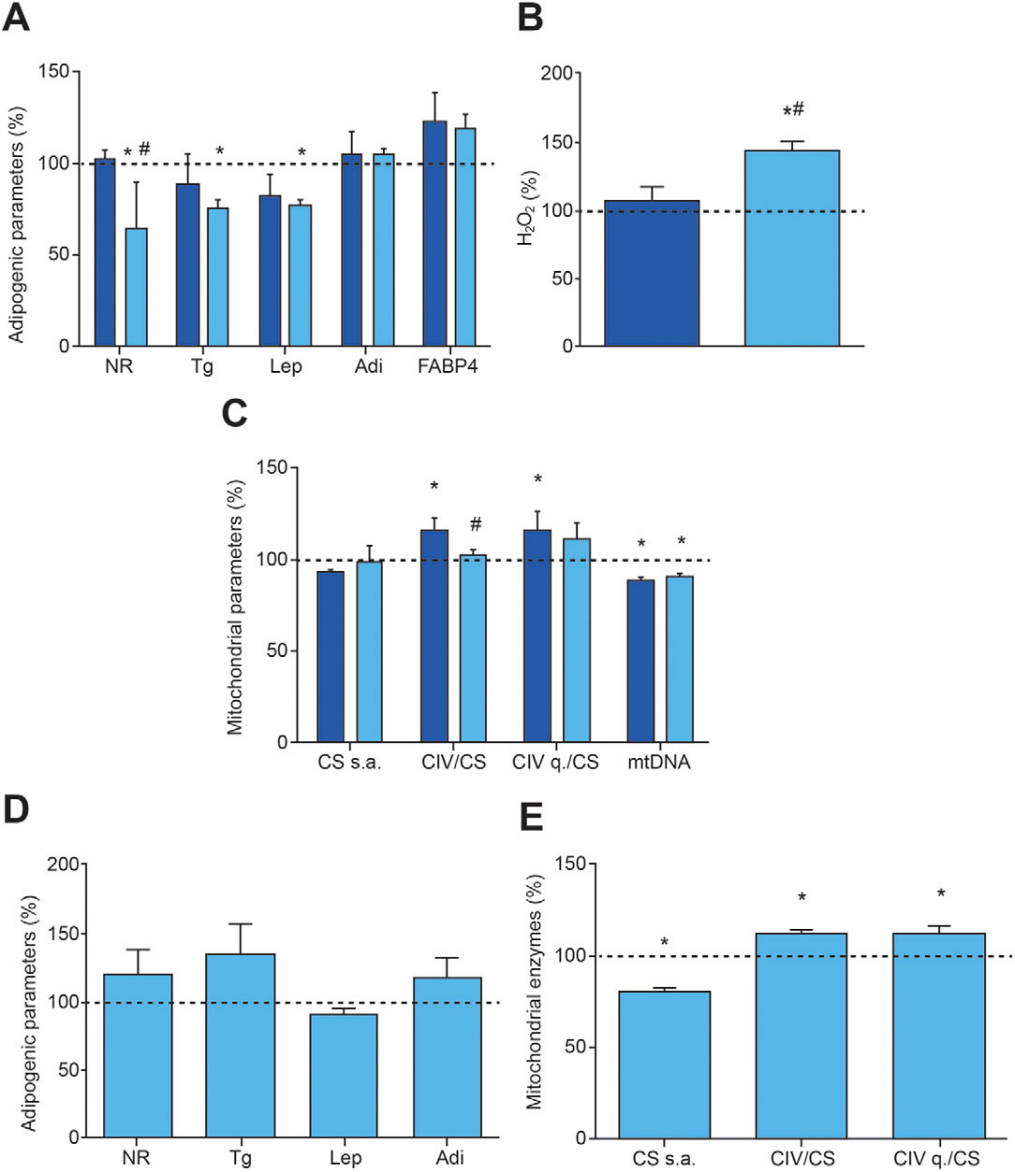
**Response of adipogenic and mitochondrial parameters to NRTIs.** Dark and light blue bars indicate treatment with 6 μM AZT and 0.1 μM ddC, respectively. Dotted lines represent levels in untreated adipocytes. (A) Adipogenic variables. *P≤0.0135, ^#^*P*=0.0234 (vs AZT). (B) H_2_O_2_ production in adipocytes. *P=0.0052, ^#^*P*=0.0077 (vs AZT). (C) Mitochondrial variables. *P≤0.0469, ^#^*P*=0.0452 (vs AZT). (D) Adipogenic variables after 0.01 μM ddC for 10 days and posterior differentiation. (E) Mitochondrial variables after 0.01 μM ddC for 10 days and posterior differentiation. *P≤0.0480.

Whereas AZT at 6 μM had no effect on intracellular or secreted markers of adipogenesis, ddC at 0.1 μM significantly decreased Nile Red staining, triglycerides and secreted leptin (Fig. 5A). Adipogenic differentiation of hASCs decreased H_2_O_2_ levels (Fig. 4M) and, consistent with its effects on adipogenesis, ddC (but not AZT) was associated with increased H_2_O_2_ levels (Fig. 5B). Neither AZT nor ddC had any effect on CS specific activity, suggesting lack of a general effect on mitochondrial biogenesis (Fig. 5C). However, as expected from inhibitors of mtDNA polymerase, mtDNA levels were significantly reduced. Additionally, AZT (but not ddC) increased CIV quantity and specific activity, normalized by CS specific activity.

ddC treatment during adipogenic differentiation provoked reduction of some adipocyte markers but had no effect on mitochondrial enzymes. However, when hASCs were treated with 0.1 μM ddC for 10 days and then differentiated in the absence of drug, they did not show any difference from untreated cells with regards to adipocyte markers (Fig. 5D), but CS specific activity was significantly decreased and CIV specific activity and quantity, normalized by CS specific activity, were significantly increased (Fig. 5E).

#### Ribosomal antibiotics: chloramphenicol and linezolid

**Fig. 6.**
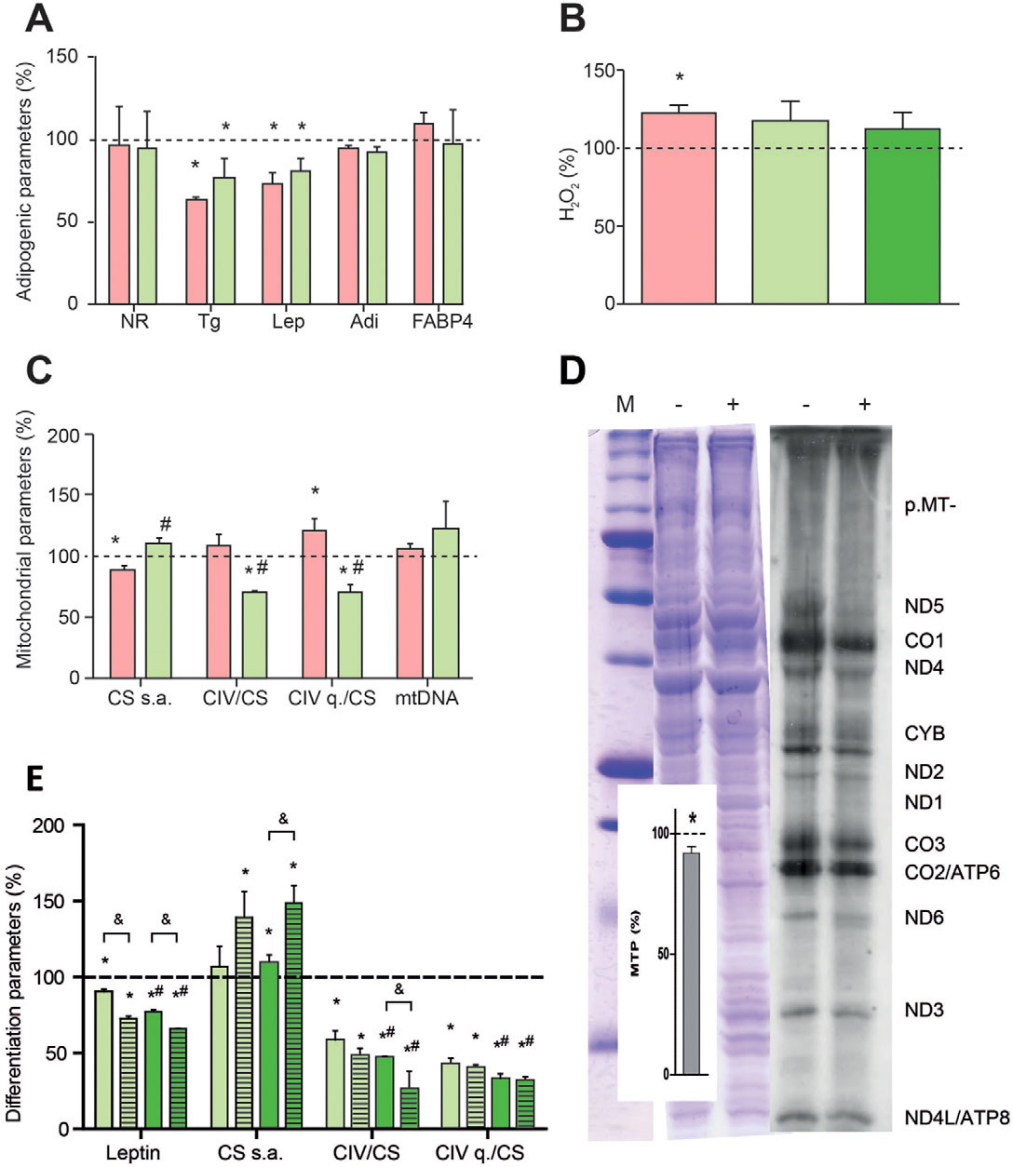
**Response of adipogenic and mitochondrial parameters to ribosomal antibiotics.** Pink and light green bars indicate treatment with 2.5 μM CAM and 30 μM LIN, respectively. Dotted lines represent levels in untreated adipocytes. (A) Adipogenic variables. *P≤0.0008. (B) H_2_O_2_ production in adipocytes. Dark green bar indicates treatment with 60 μM LIN. *P=0.0396. (C) Mitochondrial variables. *P≤0.0273, ^#^*P*≤0.0040 (vs CAM). (D) Mitochondrial protein synthesis. Representative gel showing the electrophoretic patterns of MTP (right) and loading controls (left). M, molecular weight marker; −, untreated hASCs; +, 30 μM LIN-treated hASCs. Insert: quantification of MTPs in LIN-treated hASCs. Dotted line represents the level of MTPs in untreated hASCs. *P=0.0071. (E) Response of secreted leptin and mitochondrial variables to 30 μM (light green bars) and 60 μM (dark green bars) LIN in differentiated hASCs-1 (solid bars) and hASCs-2 (striped bars). Dotted line represents levels in untreated adipocyte-differentiated hASCs-1 or hASCs-2. *P≤0.0366, ^#^*P*≤0.0329 (vs 30 μM LIN), ^&^*P*≤0.0225 (vs differentiated hASCs-1).

Because of the bacterial origin of mitochondria, some ribosomal antibiotics also inhibit mitochondrial protein synthesis. Two inhibitors of bacterial protein synthesis, chloramphenicol (CAM) and linezolid (LIN), bind the large subunit of the mitochondrial ribosome and produce myelosuppression, lactic acidosis and optical and peripheral neuropathy (Pacheu-Grau et al., 2010). Both CAM at 2.5 μM, a concentration below the lower limit of its therapeutic range (Balbi, 2004), and LIN at 30 μM, a concentration below the steady-state peak serum concentrations (Dryden, 2011), significantly decreased the amount of triglycerides and secreted leptin (Fig. 6A). However, Nile Red staining and adiponectin and FABP4 levels were not significantly affected. CAM, but not LIN at 30 or 60 μM, increased production of reactive oxygen species (ROS) (Fig. 6B). CAM, but not LIN, decreased CS specific activity (Fig. 6C). Interestingly, the ratio CIV (specific activity or quantity)/CS specific activity seemed to be altered in opposite directions by the two antibiotics, whereas mtDNA levels remained unaltered. Consistent with a negative effect of LIN on CIV quantity, a general decrease in mitochondrial protein synthesis was observed in the presence of the drug (Fig. 6D).

To rule out a particular effect of LIN on the specific hASCs used (hASCs-1), we analyzed in parallel the influence of 30 or 60 μM LIN on other hASCs (hASCs-2) derived from a different donor (Fig. 2). LIN at 30 μM provoked similar effects on hASCs-2 as observed on hASCs-1, except for CS specific activity, which was significantly increased (Fig. 6E). At 60 μM LIN, the effects on both lots of hASCs were more pronounced: the CIV/CS ratios and the levels of secreted leptin were significantly lower than at 30 μM in both cell lines (Fig. 6E). Interestingly, at 60 μM, the CIV/CS specific activities ratio and the secreted leptin were also significantly lower in hASCs-2 than in hASCs-1 (Fig. 6E). This suggests that genetic polymorphisms modify the susceptibility of hASCs to antibiotics targeting mitochondrial ribosomes.

#### Environmental toxins: tributyltin chloride

The organotin tributyltin chloride (TBTC) is a potent algicide and molluscicide. Widespread environmental contamination of marine ecosystems with tributyltins (TBT) began in the 1960s when it became ubiquitous as the active component in marine antifouling paints for ships. However, environmental contamination by TBT goes beyond aquatic ecosystems; they are also frequently used in many industrial and agricultural activities. All this has led to concern over their effects on human health. As a result, a global ban on the use of organotin-based antifouling paints was enacted from 2003 onward (Grun, 2014).

It was previously shown that TBT induces lipid droplet accumulation in human keratinocytes and adipogenic differentiation of mouse preadipocyte cells (Corsini et al., 2003; Kanayama et al., 2005). Bovine submitochondrial particles treated with TBT were found to synthesize ATP at very low rates, and it was reported that subunit p.MT-ATP6 of the OXPHOS complex V (CV, or ATP synthase) was the target site for inhibition by TBT (Matsuno-Yagi and Hatefi, 1993; von Ballmoos et al., 2004). However, these data were obtained at relatively high concentrations of the drug (Kotake, 2012). TBT concentrations in human blood have been found to range from 16.8 to 306.8 nM (Kannan et al., 1999; Whalen et al., 1999), and TBT at 10 nM can activate genomic pathways via PPARγ and retinoid X receptor alpha (RXRα) (Kanayama et al., 2005). It has also been shown recently that, depending on the exposure window, TBT can promote adipogenesis independently of PPARγ (Biemann et al., 2014). Inhibition of isocitrate dehydrogenase (IDH) at nanomolar levels has been demonstrated as a non-genomic, endocrine-disrupting mechanism (Yamada et al., 2014).

**Fig. 7.**
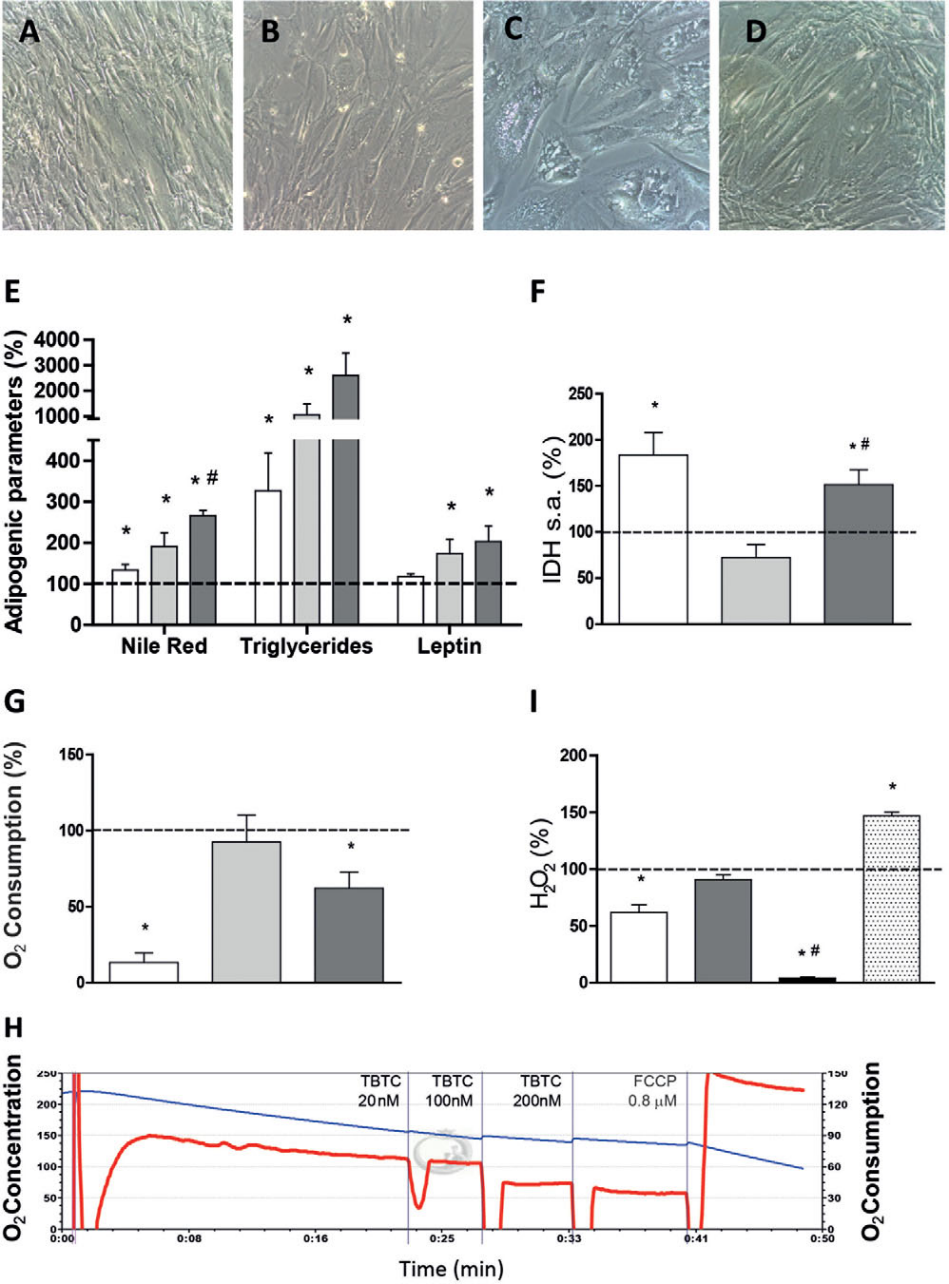
**Response of adipogenic and mitochondrial parameters to TBTC.** White, light and dark gray bars indicate 16 nM OLI-, 20 nM TBTC- and 100 nM TBTC-treated hASCs, respectively. Dotted lines represent levels in untreated hASCs. (A-D) Representative microscopy images of untreated hASCs (A), hASCs treated with 16 nM OLI (B), hASCs treated with 20 nM TBTC (C) and hASCs treated with 100 nM TBTC (D). (E) Adipogenic variables. *P≤0.0256, ^#^*P*=0.0221 (vs 20 nM TBTC). (F) IDH levels. *P≤0.0173, ^#^*P*=0.0030 (vs 20 nM TBTC). (G) Determination of oxygen consumption just after drug addition. *P≤0.0403. (H) Oxygen consumption plot. Blue and red lines represent oxygen concentration and consumption, respectively. (I) Determination of H_2_O_2_ levels after drug addition. White, gray, black and dotted bars represent 16 nM OLI-, 100 nM TBTC-, 100 nM TBTC+5 mM NAC- and 10 μM Menadione-treated hASCs, respectively. *P≤0.0021, ^#^*P*<0.0001 (vs 100 nM TBTC).

In the preliminary test for the effects of TBTC on hASCs it was recorded that another CV inhibitor, oligomycin (OLI) at 16 nM, induced similar morphological changes in cells as those observed with TBTC (Fig. 7A-D). TBTC at 20 and 100 nM significantly increased the intracellular (Nile Red, triglycerides) and secreted (leptin) markers of adipogenesis in hASCs grown for 21 days in normal, non-differentiation media (Fig. 7E and Fig. S2). Similar results were obtained for OLI at 16 nM. However, in our hands, neither TBTC nor OLI decreased IDH activity. On the contrary, TBTC at 100 nM or OLI at 16 nM significantly increased IDH activity (Fig. 7F), probably mirroring a compensatory response for increasing mitochondrial biogenesis. As expected from direct inhibitors of ATP synthase, both OLI and TBTC (at 100 nM) decreased oxygen consumption of hASCs (Fig. 7G) and the effect of TBTC was shown to be immediate (Fig. 7H). TBTC at 100 nM did not affect the levels of H_2_O_2_ but, unexpectedly, OLI was found to immediately decrease these levels (Fig. 7I). The assay was confirmed functional by addition of antioxidant *N*-acetyl-cysteine (NAC) or a superoxide-generating agent (Menadione), which decreased and increased H_2_O_2_, respectively. OLI at 4 nM was previously shown to increase H_2_O_2_ in mouse preadipocytes (Carriere et al., 2003). It remains to be seen whether the contrasting results were caused by differences in the OLI concentration used or reflect true differences between the cells from the two species.

## DISCUSSION

### Adipogenic differentiation increases OXPHOS biogenesis

In the hASCs used here, oxygen consumption, MIMP, CIV specific activity and CIV quantity per cell increased with differentiation. Thus, our results imply that adipogenic differentiation is accompanied by OXPHOS biogenesis. Oxygen consumption is also higher in differentiated than in immature primary subcutaneous preadipocytes of young adults (von Heimburg et al., 2005). Higher endogenous and uncoupled oxygen consumption is also observed after adipogenic differentiation in hMSCs and in Simpson–Golabi–Behmel syndrome subcutaneous preadipocytes (Keuper et al., 2014; Zhang et al., 2013). Mitochondrial biogenesis is increased through adipogenic differentiation of hMSCs (Tormos et al., 2011; Zhang et al., 2013). Mitochondria of adipocytes are preferentially distributed around the lipid droplets. Analagous to our findings with hASCs, *CS* and *SOD2* mRNA levels increase during adipogenic differentiation of hMSCs derived from bone marrow aspirates (Hofmann et al., 2012; Zhang et al., 2013). Moreover, the mRNA levels of cytochrome oxidase subunit 3 (p.MT-CO3) and the quantity of OXPHOS supercomplexes are strikingly increased in adipocyte mitochondria compared with undifferentiated cells (Hofmann et al., 2012). Similar results have also been obtained with cells from other species (Luo et al., 2008; Ryu et al., 2013; Shi et al., 2008; Wilson-Fritch et al., 2003). Therefore, considering that diverse differentiation protocols have been used in these reports, the collective results confirm that mitochondrial biogenesis is a common phenomenon during adipocyte differentiation.

### Inhibitors of mtDNA replication affect adipogenic differentiation

Antiretroviral therapy has been effective in lowering acquired immunodeficiency syndrome incidence among patients infected with HIV. However, NRTIs used as a component of these therapies can cause serious mitochondrial toxicity because they also inhibit mtDNA polymerase-γ. It was previously shown that NRTIs might affect OXPHOS function and adipocyte differentiation and that mtDNA genetic background (haplotype) influences the propensity for lipoatrophy in patients receiving NRTIs (Hendrickson et al., 2009). In the range of concentrations used in this study, AZT and ddC have been shown to decrease Oil Red staining both in mouse and human preadipocytes (Caron et al., 2004; Stankov et al., 2010, 2013, 2008; Walker et al., 2006). AZT at 1 μM also decreased mtDNA and p.MT-CO2 levels in mouse preadipocytes (Walker et al., 2006) but, even at 6 μM, did not have any effect on mtDNA levels or on specific activities of CIV or CS in primary human subcutaneous preadipocytes (Stankov et al., 2010). ddC at 0.2 μM decreased mtDNA and p.MT-CO2 abundance in mouse preadipocytes (Walker et al., 2006). Even though ddC at 0.1 μM decreased mtDNA levels in primary human subcutaneous preadipocytes, it did not alter CIV or CS specific activities (Stankov et al., 2010). In hASCs, ddC at 0.1 μM decreased Nile Red staining, amount of triglycerides, secreted leptin, ROS production and mtDNA levels, but did not affect the CIV/CS ratio. However, AZT at 6 μM decreased mtDNA levels and increased the CIV/CS ratio, but did not have an effect on the other parameters.

It is remarkable that NRTIs seem to affect mtDNA and lipid levels in diverse cell types from different species and using distinct concentrations and differentiation protocols. mtDNA depletion is a result of inhibition of mtDNA polymerase-γ by NRTIs. Curiously, NRTI-mediated mtDNA depletion of mouse preadipocytes is accompanied by a decrease in p.MT-CO2 levels and MIMP (Walker et al., 2006), whereas mtDNA depletion in hASCs (here) or in primary human subcutaneous adipocytes is not followed by CIV deficiency (Stankov et al., 2010). Preserved CIV activity in adipose tissue of HIV-infected patients with lipoatrophy, despite mtDNA depletion, has been previously documented (Kim et al., 2005, 2008). Thymidine kinase 2 (TK2) deficiency in humans causes mtDNA depletion syndrome (Sun and Wang, 2014). mtDNA-depleted TK2-deficient mice show reduced fat accumulation and severe reduction in leptin mRNA and circulating levels of leptin (Villarroya et al., 2011). However, CIV activity is not lowered in WAT from TK2-deficient mice, despite a significant reduction in mtDNA-encoded transcripts and moderate mtDNA depletion (Villarroya et al., 2011). It remains possible that other OXPHOS complexes are preferably affected in this model. That the mtDNA depletion directly decreases lipid accumulation is supported by the fact that treatment of mouse 3T3-L1 cells with ethidium bromide (which depletes mtDNA) decreases the intensity of Oil Red O staining (Ryu et al., 2013).

When 0.1 μM ddC was supplied for 10 days before the differentiation protocol, the parameters of adipogenic differentiation were no different to those of untreated cells. However, CIV specific activity and quantity, normalized by CS specific activity, were significantly increased. Almost the same relationship between differentiation and mitochondrial variables was observed when AZT 6 μM was provided to hASCs during the differentiation protocol. Possibly, OXPHOS compensation is able to improve differentiation factors.

### Inhibitors of mitochondrial translation affect adipogenic differentiation

It is known that some antibiotics that target bacterial ribosome also inhibit mitochondrial protein synthesis (Pacheu-Grau et al., 2010), but their potential effect on adipocyte differentiation has not been previously studied. We have shown here that ribosomal antibiotics CAM and LIN impair differentiation of hASCs, as evidenced by decreased intracellular triglycerides and secreted leptin. LIN also significantly decreased CIV/CS ratios and inhibited mitochondrial protein synthesis. However, Nile Red staining of hASCs was not decreased with CAM or LIN, which is similar to previous results on lipophilic staining of mouse preadipocytes treated with CAM (Vankoningsloo et al., 2006). Cellular fat stores primarily take the form of triglycerides, and there is some data indicating that Nile Red staining does not always correspond to quantitative triglyceride assays. In *Caenorhabditis elegans*, for example, the major fat stores are not normally stained by Nile Red, but the stain is found exclusively in lysosome-related organelles (O'Rourke et al., 2009; Zhang et al., 2010). It remains to be seen whether the observed discrepancy between triglycerides and lipophilic staining could be, at least partially, a result of the nonspecificity of stained compartments in hASCs.

In our hands, LIN diminished leptin secretion but did not affect two other adipokines (adiponectin and FABP4). A different regulation for these adipokines in response to ETC inhibition has been previously reported. Thus, leptin and adiponectin expression were decreased and increased, respectively, in mouse preadipocytes treated with the respiratory complex I (CI) inhibitor capsaicin that, similarly to LIN, also decreased triglyceride levels (Hsu and Yen, 2007). Moreover, adiponectin and leptin release was increased and decreased, respectively, in human adipocytes differentiated at 10% oxygen compared with 21% (Famulla et al., 2012). The explanation could be that leptin, adiponectin and FABP4 secretion involve distinct intracellular compartments: endosomal compartments are required for adiponectin but not for leptin secretion (Xie et al., 2008), and FABP4 is actively released via a non-classical, calcium-dependent mechanism (Schlottmann et al., 2014). However, currently there is no information on how these pathways could be selectively modulated by mitochondrial activity.

Very interestingly, for 60 μM LIN, the CIV/CS ratio was significantly lower in hASCs with mtDNA from haplogroup H10e2 (hASCs-2) than in haplogroup T2c1d1a (hASCs-1), and the same was observed with secreted leptin. We have previously found that the amount of mitochondrial translation products, the p.MT-CO1/succinate dehydrogenase complex subunit A (SDHA) ratio and the CIV/CS ratio were significantly lower after treatment with LIN in osteosarcoma 143B cybrids harboring the mtDNA m.3010A allele (Pacheu-Grau et al., 2013). This nucleotide position is located in the 16S ribosomal RNA, where LIN binds. The two hASCs types used here have the m.3010G allele, but approximately one quarter of western European individuals (mtDNA haplogroups H1 and J1) harbor the A variant. These individuals would be even more sensitive to the inhibitory effects of LIN on adipogenic differentiation. Here, the differences in CIV and secreted leptin between cells treated with different concentrations of LIN, and between hASCs-1 and hASCs-2, emphasize the importance of OXPHOS function on adipocyte differentiation that can be significantly modified by mtDNA haplotype.

### Inhibitors of OXPHOS complex activities affect adipogenic differentiation

It was long thought that the effect of TBT on adipocyte differentiation was solely through genomic pathways via PPARγ and RXRα (Kanayama et al., 2005). However, the role of TBT as PPARγ agonist in hASCs is questionable. It has been shown that hASCs do not express PPARγ before adipogenic differentiation. The same seems to be true in mouse mesenchymal stem cells (mMSCs) C3H10T1/2. Thus, during undifferentiated growth, PPARγ2 is rarely expressed. In C3H10T1/2, exposure to 100 nM TBT during undifferentiated growth increased expression of the adipogenic marker genes *FABP4* and *PPARγ2*, and subsequently increased triglyceride levels. However, treatment with the PPARγ2 agonist rosiglitazone had no significant effect on subsequent adipogenic differentiation. Moreover, silencing of *PPARγ* had no additional impact on the regulation of cell fate markers, over that already observed with TBT. These results suggest that the TBT-induced adipogenic mechanisms act independently of PPARγ2 during undifferentiated growth (Biemann et al., 2014) and, in part, during the induction of differentiation in mMSCs (BMS2) (Yanik et al., 2011).

It has been shown that the proton channel of the F_1_F_0_ ATP synthase is a target of TBT, possibly through formation of Sn-O bonds at crucial, conserved amino acid residues (Grun, 2014). This leads to inhibition of proton flow and reduction in intracellular ATP levels (Matsuno-Yagi and Hatefi, 1993; von Ballmoos et al., 2004). Accordingly, our results suggest that TBTC induces adipogenesis by an OXPHOS-mediated mechanism. Moreover, 6-day exposure to 25 nM TBT provoked a significant decrease in ATP levels and loss of lytic function in human natural killer cells (Dudimah et al., 2007). Inhibition of OXPHOS CV could also explain TBT-dependent isocitrate accumulation (Yamada et al., 2014), as NAD^+^ required for its oxidation would not be regenerated by an inhibited ETC. It was reported that OXPHOS CV inhibitor OLI at 8 nM induces triglyceride accumulation in 3T3-L1 cells (Vankoningsloo et al., 2005), which is similar to our results.

Published data on the potential role of inhibition of other OXPHOS complexes are somewhat contradictory. Rotenone, a CI inhibitor, decreased Oil Red O staining and adiponectin mRNA levels of adipocyte-differentiated hMSCs (Zhang et al., 2013) but induced lipid accumulation in hASCs (Kim et al., 2014). Antimycin A, a complex III (CIII) inhibitor, decreased adipogenic differentiation of 3T3-F442A mouse preadipocytes (Carriere et al., 2004) but again induced adipogenesis in hASCs (Kim et al., 2014). Additionally, it has been shown that antimycin A, as well as stigmatellin and myxothiazol (other CIII inhibitors), can induce triglycerides in 3T3-L1 cells (Vankoningsloo et al., 2006, 2005). Thus, more detailed work on different classes of ETC inhibitors are required to evaluate their adipogenic versus antiadipogenic effects on various types of cells capable of adipogenic differentiation.

### Conclusions

Here, we have shown that the mitochondrial OXPHOS system is important for regulation of adipogenesis in hASCs and that its inhibition at the level of mtDNA replication (NRTIs), organelle protein synthesis (antibiotics) or function (TBTC) can either inhibit or promote adipogenesis *in vitro*. Because important OXPHOS components are mtDNA-encoded and OXPHOS capacity is affected by mtDNA haplogroup (Gomez-Duran et al., 2010, 2012), genetic variability in mtDNA could affect adipocyte differentiation and related phenotypes such as obesity (Knoll et al., 2014; Nardelli et al., 2013; Yang et al., 2011).

In the fight against infectious diseases, as well as in agricultural pest control, therapies are based on the selective toxicity of the used drug or xenobiotic against the non-desired agent. In this light, the human OXPHOS system can be seen as the unintended off-target of some drug treatments and responsible, at least in part, for adverse events associated with a large array of compounds (Wallace, 2008), including those related to adipogenic differentiation. As previously commented, mtDNA haplogroups modify the lipoatrophy propensity of patients being treated with NRTIs (De Luca et al., 2012; Hendrickson et al., 2009; Hulgan et al., 2011). mtDNA haplogroups can also define cell susceptibility to inhibitors of mitochondrial protein synthesis (Pacheu-Grau et al., 2013). These observations suggest that the mtDNA genotype is a risk factor for lipoatrophy in patients treated with OXPHOS xenobiotics.

If the potential side effects (such as altered adipocyte differentiation) of a particular pharmacological intervention are known, they can be monitored and possibly prevented. However, human exposure to a myriad of other OXPHOS xenobiotics that affect adipocyte differentiation is unknown and uncontrolled. Thus, as the obesogen hypothesis postulates (Grun, 2014), environmental chemical pollutants such as TBT that promote adipose-dependent weight gain might lead to obesity and metabolic syndrome.

## MATERIALS AND METHODS

### Chemicals

All reagents used were of research or cell culture quality. AZT and ddC, which affect mitochondrial function and adipogenic differentiation; CAM, LIN and OLI, which affect mitochondrial function; and TBTC, which affects adipogenic differentiation, were purchased from Sigma-Aldrich (St Louis, MO, USA).

### Cells, growth and differentiation conditions

StemPro^®^ Human Adipose-Derived Stem Cells (#R7788-115, Invitrogen™, Life Technologies™) derive from human adipose tissue collected during liposuction procedures and were cryopreserved at passage 1 from primary cultures. Each lot of hASCs originates from a single donor of human lipoaspirate tissue, and the two lots used here are named hASCs-1 and hASCs-2. hASCs were generally grown in MesenPRO RS™ medium (Invitrogen™, Life Technologies™), containing 5 mM glucose and 2% fetal bovine serum complemented with MesenPRO RS™ growth supplement and 2 mM L-glutamine prior to use. To avoid undesired phenotypic effects, cells were grown in the absence of antibiotics (Llobet et al., 2015). The doubling times were determined using the Z2 Beckman Coulter. Initially, 2×10^4^ cells per well were seeded in a six-well plate. Three replicate growth curves were performed and cells counts were performed in duplicate at each time point (0, 24, 48, 72 and 96 h). For the hypoxia treatment, cultures were transferred to an H35 Hypoxystation (Don Whitley Scientific), where the incubation proceeded in atmosphere containing 5% CO_2_ and 3% O_2_ balanced with nitrogen.

To induce adipogenic differentiation, confluent hASCs were incubated for 21 days with StemPro^®^ Adipogenesis Differentiation Kit (Invitrogen™). Because this differentiation medium contains 25 mM glucose, the glucose concentration of the control culture medium (MesenPRO RS™) for undifferentiated cells was also raised to 25 mM in experiments where undifferentiated and differentiated cells were compared.

For osteogenic differentiation, hASCs were grown to 50% of confluence and then incubated for 14 days with StemPro^®^ Osteogenesis Differentiation Media (Invitrogen™). For assessment of calcium deposition, cells were washed with phosphate-buffered saline (PBS), fixed for 30 min in 4% paraformaldehyde at room temperature and washed with distilled water. Samples were incubated for 5 min at room temperature in 30 mM Alizarin Red in distilled water, washed and visualized using an inverted microscope.

For chondrogenic differentiation, 80% confluent hASCs were harvested and micromass cultures were generated by seeding the center of 96-well plate wells with 5 μl droplets from a cell suspension containing 1.6×10^7^ cells/ml. After 2 h, warmed StemPro^®^ Chondrogenesis Differentiation Medium (Invitrogen™) was added and the micromass cultures were incubated for 14 days. The differentiated cell aggregates were fixed with 4% paraformaldehyde for 30 min at room temperature and stained with 1% Toluidine Blue in 70% ethanol, which detects glycoproteins in the extracellular matrix. An inverted microscope was used for imaging.

### Metabolic markers

Glucose concentration was measured by an enzymatic colorimetric GOD-POD method with the Glucose Liquid reagents (Química Clínica Aplicada S.A., Tarragona, Spain) following a previously described protocol (Trinder, 1969). Briefly, the cells under study were seeded, supernatant samples were collected three times per day (6 h intervals) and absorbance was measured at 510 nm using the NovoStar MBG Labtech microplate instrument.

Lipid accumulation was assessed by Oil Red O staining. Briefly, cells were washed with PBS, fixed with 4% paraformaldehyde for 30 min at room temperature and incubated for 30 min at 37°C in freshly diluted and filtered 16 µM Oil Red O solution in isopropanol. Alternatively, intracellular lipids were stained with the hydrophilic stain Nile Red that, when partitioned in a hydrophobic environment, becomes fluorescent. For quantitative determination of Nile Red fluorescence, the NovoStar MBG Labtech microplate instrument was used (Ex: 485 nm/Em: 572 nm). Images were acquired either with a DMIL inverted microscope (Leica) or FLoid Cell Imaging Station (Life Technologies™).

Adipogenesis Detection Kit (Abcam) was used to quantify triglyceride accumulation in cells according to the manufacturer's instructions. In this assay, triglycerides are solubilized and hydrolyzed to glycerol, which is subsequently oxidized to convert the probe to generate color (λ_max_=570 nm). A NovoStar MBG Labtech microplate instrument was used for measurements. For quantitative determination of adiponectin, FABP4 and leptin in cell culture supernatants, Human Adiponectin ELISA kit (Millipore), FABP4 Human ELISA kit (Symansis) and Leptin Human ELISA Kit (Abcam) were used. Media were centrifuged for 5 min at 1400 rpm (Beckman Coulter Allegra X-22, SX4250 rotor) and supernatants diluted for use in the quantification reaction. Concentrations were determined according to the manufacturer's instructions.

For immunocytochemistry, the cultured cells were fixed with 4% paraformaldehyde for 15 min at room temperature and permeabilized with 0.1% Triton X-100 for 10 min. After blocking with 0.1% bovine serum albumin, the washed cells were incubated for 1 h at room temperature with a primary antibody against FABP4 (Invitrogen). Subsequently, the cells were incubated with fluorescence-labeled secondary Alexa Fluor^®^ 594 (Molecular Probes) at room temperature for 30 min, protected from light. The cells were further incubated with 1 µM 4′,6-diamidino-2-phenylindole (DAPI) for nuclear staining. Between incubations, samples were washed with PBS containing 0.05% Tween.

### Electron microscopy

For ultrastructural analysis, hASCs were seeded in Permanox^®^ chamberslides (NUNC), fixed with 2.5% glutaraldehyde for 2 h at 4°C and maintained in phosphate buffer supplemented with 0.05% sodium azide. Post-fixation was carried out using 1% OsO_4_ for 2 h. The cells were dehydrated in a graded series of ethanol up to absolute. The specimens were then passed through different mixtures of ethanol and araldite (3:1, 1:1, 1:3) and embedded in pure araldite. After 3 days polymerization at 70°C, ultrathin sections were cut and stained following a previously published protocol (Reynolds, 1963). The sections were examined with a JEOL 1010 transmission electron microscope using a Gatan Bioscan camera and the Digital Micrograph software.

### Analysis of mitochondrial function

To measure mitochondrial content, mitochondria were labeled using the mitochondria-specific dye MitoTracker^®^ Red (Molecular Probes™), according to the manufacturer's protocol. The final dye concentration was 100 nM and the incubation time was 30 min at 37°C prior to visualization. Fluorescent microscopy was performed on live cells using a FLoid Cell Imaging Station (Life Technologies™). MIMP was measured in triplicate in three independent experiments using the Mito-ID Membrane Potential Cytotoxicity kit (ENZO^®^) following the manufacturer's instructions. MIMS, based on the quantity of cardiolipin, was measured three times in three independent experiments using nonylacridine-orange (Sigma) (Petit et al., 1992). H_2_O_2_ production was measured in triplicate using 2′,7′-dichlorofluorescin diacetate (Sigma) as described previously (Carter et al., 1994), with minor modifications. A Beckman Coulter Cytomics FC500 cytometer was used for measurement of intracellular fluorescence, and Weasel software was used for flow cytometry data analysis.

Oxygen consumption was analyzed using the high-resolution oxygraph OROBOROS^®^ (Oroboros Instument, Innsbruck, Austria). Exponentially growing cells were collected by trypsinization, then washed, counted and resuspended at 1×10^6^ cells/ml in DMEM. Endogenous, leaking (with OLI added at 16 nM) and uncoupled (with FCCP added at 0.8 µM) respiration analyses were performed. To correct for oxygen consumption not due to the ETC, inhibition of mitochondrial respiration by KCN was performed. Each condition was analyzed three times. Respiration was measured at 37°C, with chamber volumes set at 2 ml. The software DatLab (Oroboros Instrument, Innsbruck, Austria) was used for data acquisition at 1 s time intervals, as well as for data analysis (Gnaiger et al., 1995).

The enzymatic activities of OXPHOS CII and CS and their protein levels were assayed following previously described protocols (Faloona and Srere, 1969; King, 1966; Tzagoloff et al., 1967). CIV activity and levels were determined using the Complex IV Human Specific Activity Microplate Assay Kit (Mitosciences, Abcam^®^) according to the manufacturer's instructions. All enzyme determinations were performed in triplicate in at least three independent experiments using a NovoStar MBG Labtech microplate instrument. IDH activity was determined using the commercial Isocitrate Dehydrogenase Colorimetric Assay Kit (Abcam^®^), according to the manufacturer's instructions. Briefly, 1×10^6^ hASCs grown in DMEM were lysed in 200 µl of provided assay buffer. The lysate was centrifuged at 13,000 ***g*** for 10 min and the cleared supernatant used for the assay. NAD^+^ was used as substrate for the NAD-IDH assay. A NovoStar MBG Labtech microplate instrument was used for the measurements.

Mitochondrial protein synthesis was analyzed as described previously (Chomyn, 1996), with minor modifications. Electrophoresis was performed with a Protean II xi system (Bio-Rad). As a loading control, we dyed the gel for 15 min with fixing solution (30% methanol, 10% acetic acid) plus 0.025% Coomassie Blue (Brilliant Blue R, Sigma). The gel was washed several times with 50% methanol, 10% acetic acid solution and left overnight in fixing solution. Finally, the gel was treated for 20 min with Amplify solution (Amersham), dried and used for autoradiography. The band intensities from appropriate exposures of the fluorograms from three independent gels were quantified by densitometric analysis with the Gelpro analyzer v4.0. Three bands, corresponding to the upper, middle and lower parts of the gel, were selected for quantification.

### Genetics characterization and gene expression analysis

For molecular cytogenetic analysis, cells were exposed to colchicine (0.5 µg/ml) for 4 h at 37°C and harvested routinely. Metaphases were prepared following a conventional cytogenetic protocol for cells fixed in methanol:acetic acid (3:1). Approximately 20 metaphase cells were captured and analyzed for each cell line. The genetic fingerprints of the cells were performed with the AmpFLSTR^®^ Identifiler^®^ PCR Amplification Kit (Life Technologies). The mtDNA sequences were obtained using the BigDye Terminator v3.1 Cycle Sequencing Kit (Applera Rockville, MD, USA) and an ABI Prism 3730xl DNA analyzer (Applied Biosystems, Foster City, CA, USA). To locate mutations, the revised Cambridge Reference Sequence (rCRS) was used (GenBank NC_012920) (Andrews et al., 1999).

The mtDNA content was measured by the real-time quantitative reverse transcription-polymerase chain reaction (RT-qPCR) method using an Applied Biosystems StepOne™ Real-Time PCR System Thermal Cycling Block, as described elsewhere (Marcuello et al., 2005). The mtDNA levels were determined in triplicate in three independent experiments.

To assess mRNA levels, total RNA was isolated from exponentially growing cells using a NucleoSpin^®^ RNA II kit (Macherey-Nagel) according to the manufacturer's protocol. Total RNA (1 µg) was reversed-transcribed with the Transcriptor First Strand cDNA Synthesis Kit (Roche), using the manufacturer's conditions. The levels of *CD90/THY1* and *CD105/ENG*, *FABP4*, *PGC1α*, *PPARγ*, *VEGF*, *PFKL* and *SOD2* mRNAs and cytochrome oxidase subunit 1 (p.MT-CO1), 12S ribosomal RNA and NADH-ubiquinone oxidoreductase chain 6 (p.MT-ND6) mitochondrial RNA were determined in triplicate in three independent experiments by RT-qPCR using the One-Step Real-Time system (Applied Biosytems). The expression levels were normalized using the 18S ribosomal RNA. The ΔCt method was used to calculate fold expression. StepOne software version 2.0 (Applied Biosystems) was used for data analysis.

### Statistics

The statistical package StatView 6.0 was used to perform all the statistics. Data for mean, standard deviation and number of independent experiments or replicates are presented. The Kolmogorov–Smirnov test was used to check the normal distribution. The unpaired two-tailed *t*-test was used to compare parameters. *P* values lower than 0.05 were considered statistically significant.
